# Students’ Consumption of Beverages and Snacks at School and Away from School: A Case Study in the North East of Italy

**DOI:** 10.3389/fnut.2015.00030

**Published:** 2015-10-07

**Authors:** Carmen Losasso, Veronica Cappa, Marian L. Neuhouser, Valerio Giaccone, Igino Andrighetto, Antonia Ricci

**Affiliations:** ^1^Risk Analysis and Public Health Department, Istituto Zooprofilattico Sperimentale delle Venezie, Legnaro, Italy; ^2^Division of Public Health Sciences, Fred Hutchinson Cancer Research Center, Seattle, WA, USA; ^3^Animal Medicine, Production and Health, University of Padua, Padua, Italy

**Keywords:** food habits, BSQ, healthy eating, beverages and snacks consumption

## Abstract

In North-East Italy (the Veneto region), several public school nutrition policies have been developed to reduce the consumption of high-caloric snacks and beverages. However, little is known about whether the policies actually influence students’ dietary behaviors. In order to address this point, a multi-center cross-sectional survey of 691 Italian students was conducted. Students completed the Beverage and Snack Questionnaire, which assesses the consumption of beverages and snacks at school and out of school. Three-level Poisson Models with random intercept with students (level 1 units) nested into classroom (level 2 units), and nested into schools (level 3 units), were used to examine the influence of the school setting vs. the out of school environment (independent variable) on students’ consumption of sweet beverages, snacks, milk-based beverages, low-carbohydrate drinks, fruit, and vegetables (dependent variable) (*p* ≤ 0.05). The results showed a significantly higher consumption of sweet beverages, snacks, milk-based beverages, low-carbohydrate drinks, fruit, and vegetables out-of-the school, suggesting a school-protective association Thus, the policies aimed to limit or deny access to unhealthy foods in the school environment may play an important role in promoting more healthful dietary patterns for school children. Additional studies should be conducted to compare students’ dietary behaviors between schools with nutrition policies to those without nutrition policies.

## Introduction

According to the European Union (EU) Commission, in the last three decades, the prevalence of overweight and obesity in the EU population has risen dramatically, particularly among children. In 2006, it was estimated that 30% of children were overweight (1). The alarming trend in the prevalence of overweight and obesity is symptomatic of a worsening trend in diets and a low physical activity levels across the EU population. In Italy, the results of “*OKKIO alla Salute*,” the national surveillance program on overweight and obesity in children between 6 and 10 years of age, revealed that in 2014 about 33% of children were overweight or obese. This study also showed that parents underestimated the magnitude of the obesity problem, which could also be associated with a lack of understanding of the health consequences of childhood obesity (2, 3). Poor lifestyle habits are expected to increase the future risk of chronic diet and obesity-related conditions, while also imposing a negative influence on both quality of life and life expectancy.

Overweight commonly appears early in life and continues to adulthood (4), causing a serious health burden during and beyond childhood (5). Adolescence, with its rapid changes in body composition (4) and food habits, coincides with the transition from the parents’ direct control over food consumption to a strong influence from the peer-related environment (6).

Several studies have examined the association of diet composition with childhood obesity (7–9). Many of these studies have shown that energy-dense snacks, such as high-fat salty snacks, candies, and sugar-sweetened beverages are “*obesogenic*” foods. These foods have several characteristics, which contribute to poor diet quality and impede maintenance of a healthy weight. First, snacks and sugar-sweetened beverages are major dietary sources of calories and saturated fat (10, 11). Second, in addition to contributing to excess calories and fat, intake of energy-dense nutrient-poor snacks and beverages may have a direct impact on body composition via modification of the homeostatic control of eating behavior. This disruption may lead to further overeating and excess energy intake (12–14). Third, since *obesogenic* foods tend to also be highly palatable, there is often uncontrolled consumption (15) in the context of short and unstructured eating episodes (i.e., “snacking”), which is also associated with a lack of self-awareness of the quantity of food consumed. Fourth, consumption of snacks and sugar-sweetened beverages, particularly among overweight and obese adolescents, is commonly associated with other *obesogenic* behaviors, such as meal skipping, especially breakfast, and/or physical inactivity (16–18).

A specific link between the high intake of liquid carbohydrates through sugar-sweetened beverages consumption and the risk of overweight and obesity in children has been demonstrated as well (19), suggesting that drinking liquid carbohydrates may provoke a lack of dietary compensation compared to that observed by consuming similar amounts of solid carbohydrates (20). Therefore, sugar-sweetened beverage consumption may lead to an increase in the total energy intake since liquid sugar may have less capacity to induce satiety. Additionally, sugar-sweetened beverage consumption may not be compensated by reduced caloric ingestion from other sources. This has been documented for coffee, alcoholic beverages, fizzy drinks, fruit juice, and milk (21).

However, the results obtained from a large survey conducted on a cohort of Italian students who reported high consumption of sugar-free soft drinks and/or table-top sweeteners allowed the authors to conclude for a low risk of excessive intake of intense sweeteners in the considered sample (22).

Multiple strategies are needed both to improve eating habits and to lower the prevalence of childhood and adolescent obesity in the EU. One potentially effective approach is to create and implement food policies in the school environment that would be designed to limit access to unhealthy snacks and beverages. A modest amount of research to date suggests that the availability of obesogenic foods in schools is associated with less healthy food preferences and unhealthy food choices (23, 24).

In North-East Italy (the Veneto region), several public school nutrition policies have been implemented on behalf of the Veneto Region Health Authorities (25, 26). Moreover, in order to regulate and standardize the quality of school food and the school meals’ nutritional composition, the “Regional guidelines for school catering” have been published and their use has been adopted by schools via the ratification of the Regional Directive 475/2008.

These policies primarily focus on reducing the consumption of high-caloric snacks and beverages in the school environment, but little is known about whether the policies actually influence students’ dietary behaviors.

Therefore, our overall objective was to investigate students’ consumption of energetically dense snacks and beverages in two different environmental contexts (at school and away from school). These questions are of pivotal importance to help determine whether future interventions should be conducted in the home or school setting. This study was not designed as a policy evaluation, but rather to provide data on current consumption habits and whether restricting access to certain snacks and beverages in one setting (school) is associated with lower consumption than a setting where access is not restricted (away from school).

## Materials and Methods

### Study design

We conducted a multi-center cross-sectional survey of the dietary behaviors of 691 Italian students. Study participants were primary and secondary school students, aged 6–15 years, attending 9 public schools of the Veneto Region, in the North East of Italy. The participating schools were selected by the Regional School Coordination Office based on the following criteria: (i) prior endorsement of the “*Regional guidelines for school catering*” (Veneto Region, D.G.R. 31.12.2001 n. 3883); (ii) upholding a ban on vending machines and cafeterias for one scholastic year; (iii) implementation of at least one of the regional or national educational programs for “*healthy eating*” in collaboration with the competent local health authority; (iv) involvement in the national health program “*frutta nelle scuole*”^1^ implemented by Italian Ministry of Agriculture, aimed at encouraging students to consume seasonal fruit at break-time; (v) involvement in the national health program “*forchetta e scarpetta*”^2^ implemented by the Italian Ministry of Education; and (vi) provision of two daily meals: a morning snack and the lunch for all students. In order to ensure the presence of both primary and secondary schools, the study was conducted in three secondary schools and six primary schools randomly chosen from the five most populated provinces of the Region (Treviso, Padua, Verona, Vicenza, and Venice) among those eligible who voluntarily accepted the invitation.

All data collection took place between November 2012 and March 2013.

The local ethics committee approval was not required according to the Istituto Zooprofilattico Sperimentale delle Venezie guidelines.

### Questionnaire and outcomes

The objectives of the study were met by asking all students to complete the self-administered Beverage and Snack Questionnaire (BSQ), which assesses the consumption of beverage and snacks both at school and out of school. Parents approved the experimental design and signed an informed consent. The BSQ is a cost-effective, validated 19-item, self-administered instrument (27) that was originally designed to assess the effectiveness of school nutrition policies that limit access to sugar-sweetened beverages, savory snacks, and sweets. The BSQ was translated into Italian for this study. The 19 questions elicited information on consumption of (i) sweet beverages (fruit drinks, sport drinks, regular soda, and energy drinks), (ii) snacks (low-fat potato chips, regular potato chips, other salty snacks, candies, breakfast pastries, cookies, pies, cakes, low or non-fat frozen dessert, and regular ice-creams and milkshakes), (iii) milk-based beverages (low-fat milk and regular milk and yogurt), (iv) low-carbohydrate drinks (flavored water, diet soda), (v) fruits and vegetables (100% fruit juices, vegetables, and fruit). Seven response options were provided that ranged from “less than once/week” to “5–6 times/day.” Separate questions distinguish the frequency of consumption of the snacks and beverages by location. The questionnaire is available on request. In addition, information on students’ demographic characteristics (school, classroom, age, gender, ethnic group) was collected.

### Statistical analyses

The overall goal of the statistical analysis was to compare the consumption of beverages and snacks in two different environments: school and away from school. These comparisons could then provide useful information about the effectiveness of the school-based policies that influence the students’ dietary behaviors.

In the absence of any effect exerted by the school, it could be supposed that beverages and snacks consumption would be comparable, as participants were the same persons living in two different contexts. On the contrary, in case of any effect due to the scholastic policies, a difference between students’ consumption of the investigated food and beverages between the two different environments should be envisaged. In other words, a difference in the consumption might be due to bad/good habits in one of the two environments.

To address this point, we applied statistical tests designed to be applied for dependent samples and mixed models for repeated measures (28) as we repeated the same measure (for example, consumption of fruit juice) in two different environments (“domestic” and “scholastic”) for each student.

Prior to data analysis, all survey responses were entered into an electronic database (Access 2009, Microsoft Corporation, Redmond, WA, USA), and extensive QC was performed by comparing the original questionnaires with the database values.

We first summarized categorical data as counts with percentages or as a median with interquartile difference (IQD) and we tested for differences between gender and age/ethnic group using Pearson chi-squared test. Next, differences between students’ intake of beverages and snacks at school and away from school were compared by the Wilcoxon signed-rank test. To evaluate the influence of the school setting vs. the away from school ones (independent variable) on students’ consumption of sweet beverages, snacks, milk-based beverages, low-carbohydrate drinks, fruit, and vegetables (dependent variables), three-level Poisson Models with random intercept using students (level 1 units) nested into classroom (level 2 units), and nested into schools (level 3 units), were used.

Separate models were performed for each considered food (outcome), adjusting for age [≤8 vs. (8–11), >11 years], gender (female vs. male), and ethnic group (Caucasian vs. non-Caucasian). The obtained estimates described the differences of the outcomes due to different contribution of the independent variables in terms of incident risk ratio (IRR), with a relative confidence interval of 95% (95% CI). Regarding Fruit Juice consumption outcome, for example, and environment (at school out of school, independent variable), the IRR is interpreted as number of times the consumption of fruit juice is less or more out of school than at school. That is, if IRR is >1 and statistically significant (*p* ≤ 0.05), then 95% CI does not include 1, which means that children consumed IRR-times more fruit juice away from school than at school and it is possible to assert the effectiveness of the intervention; on the contrary, if IRR is <1 and statistically significant (*p* ≤ 0.05), then 95% CI does not include 1; this means that children consumed IRR-times less fruit juice away from school than at school, and the intervention is not effective.

Questionnaires with information on gender, age, or ethnic group missing were excluded from the mixed model analyses (*n* = 8).

All statistical analyses were performed using Stata Statistical Software: Release 12. All tests were two-sided with statistical significance set at *p* ≤ 0.05.

## Results

### Sample characteristics

Table 1 gives the demographic characteristics of the study sample. Briefly, the questionnaire was administered to 691 school students; 363 (52.5%) were males and 328 (47.5%) were females; their median age was 9 years (IQD 8–11). The vast majority of the participants were Caucasians (93.6%). The sample was further stratified into three age groups, which parallels the age distribution of three different didactic cycles of the Italian compulsory education: ≤8 (34.0%), (8–11) (45.7%), and >11 (20.3%).

**Table 1:** **Sample characteristics**.

	Males	Females	Total	p*
***n***	328 (52.5%)	363 (47.5%)	691	
**Age (years)**				
≤8	117 (32.2%)	118 (36.0%)	235 (34.0%)	0.06
(8–11)	181 (49.9%)	135 (41.2%)	316 (45.7%)	
>11	65 (17.9%)	75 (22.9%)	140 (20.3%)	
**Ethnic group**				
Caucasian	344 (94.8%)	303 (92.4%)	647 (93.6%)	0.20
Other ethnic groups	19 (5.2%)	25 (7.6%)	44 (6.4%)	

*Pearson chi-square test to evaluate the difference between gender and class of age/ethnic group.

### Sweet beverage consumption assessment

Table 2 compares the frequency of consumption of the 19 food and beverage items and compares the at school and out-of school consumption estimates. For each item listed, there was negligible reported consumption of sugar-sweetened beverages and sweet and salty snacks at school. Consumption of these foods, if it did exist, happened outside the school setting.

**Table 2:** **Frequency of consumption [median (IQD)] of selected foods and beverages at school and out of school**.

	At school	Out of school	p*
**Sweet beverages**			
Fruit drinks	0 (0–4)	4 (0–5)	<0.001
Sport drinks	0 (0–0)	0 (0–1)	<0.001
Regular soda	0 (0–0)	4 (0–5)	<0.001
Energy drinks	0 (0–0)	0 (0–0)	<0.001
**Snacks**			
Low-fat potato chips	0 (0–0)	0 (0–4)	<0.001
Regular potato chips	0 (0–0)	2 (0–4)	<0.001
Other salty snacks	0 (0–4)	2 (0–4)	<0.001
Candies	0 (0–0)	4 (0–5)	<0.001
Breakfast pastries	0 (0–0)	0 (0–4)	<0.001
Cookies, pies, and cakes	0 (0–0)	4 (0–5)	<0.001
Low or non-fat frozen dessert	0 (0–0)	0 (0–4)	<0.001
Regular ice-cream and milkshakes	0 (0–0)	2 (0–4)	<0.001
**Milk-based beverages**			
1.5% or non-fat milk	0 (0–0)	2 (0–4)	<0.001
Regular or 3.0% milk	0 (0–0)	1 (0–4)	<0.001
**Water drinks**			
Flavored water	0 (0–0)	0 (0–4)	<0.001
Diet soda	0 (0–0)	0 (0–4)	<0.001
**Fruits and vegetables**			
100% fruit juices	0 (0–0)	3 (0–5)	<0.001
Vegetables	0 (0–4)	3 (2–5)	<0.001
Fruits	1 (0–4)	3 (2–5)	<0.001

*Differences between the two different environments for each question (outcome) were assessed by Wilcoxon signed-rank test for dependent samples (a child reported consumes at school and out of school).

Table 3 provides the results from the three-level Poisson Models. These results show that students consumed fruits drinks twice as frequently in the out of school setting than in the scholastic one (IRR 2.1, 95% CI 1.9–2.2). Sport drinks were consumed 5.7 times more often out of school (95% CI 4.7-6.9) than at school, with males consuming more often than females (IRR 0.6, 95% CI 0.4–0.99), and students aged 8–11 years being consumers more frequently than their youngest colleagues (IRR 3.9, 95% CI 1.7–8.9). Regular soda and energy drinks were consumed more frequently in the out of school setting (IRR 10.7, 95% CI 9.1-12.5; IRR 4.4, 95% CI 3.2-6.1 respectively), and by students aged more than 11 years compared with the younger ones (IRR 1.7, 95% CI 1.3–2.2 for regular soda and IRR 11.2, 95% CI 1.04–119.5 for energy drink). Non-Caucasian students had similar habits as Caucasian students with regard to sweet beverage consumption, with the exception that non-Caucasians consumed regular soda, on average, 1.3 times more often than Caucasians (95% CI 1.0–1.8; *p* = 0.05).

**Table 3:** Differences* in the outcome scores at school and out of school, in gender, class of age, and ethnicity group.

Outcomes	Environment (out of school vs. at school)	Gender (females vs. males)	Age [(8–11) vs. ≤8]	Age (>11 vs. ≤8)	Ethnicity group (not-Caucasian vs. Caucasian)
**Sweet beverages**					
Fruit drinks	**2.1 (1.9–2.2); *p* < 0.001**	0.98 (0.9–1.1); *p* = 0.78	1.1 (0.8–1.3); *p* = 0.61	**1.4 (1.03–1.9); *p* = 0.03**	1.2 (0.97–1.6); *p* = 0.09
Sport drinks	**5.7 (4.7–6.9); *p* < 0.001**	**0.6 (0.4–0.99); *p* = 0.047**	**3.9 (1.7–8.9); *p* = 0.001**	2.4 (0.9–6.6); *p* = 0.09	2.3 (0.9–6.1); *p* = 1.0
Regular soda	**10.7 (9.1–12.5); *p* < 0.001**	0.9 (0.8–1.02); *p* = 0.10	**1.3 (1.1–1.7); *p* = 0.01**	**1.7 (1.3–2.2); *p* < 0.001**	**1.3 (1.0–1.8); *p* = 0.05**
Energy drinks	**4.4 (3.2–6.1); *p* < 0.001**	0.6 (0.2–1.4); *p* = 0.23	3.2 (0.5–20.2); *p* = 0.22	**11.2 (1.04–119.5); *p* = 0.046**	3.9 (0.8–18.9); *p* = 0.09
**Snacks**					
Low-fat potato chips	**3.5 (3.1–3.9); *p* < 0.001**	0.9 (0.7–1.2); *p* = 0.45	1.5 (0.9–2.7); *p* = 0.13	**2.1 (1.0–4.4); *p* = 0.051**	1.2 (0.6–2.2); *p* = 0.59
Regular potato chips	**4.0 (3.5–4.4); *p* < 0.001**	0.8 (0.7–1.0); *p* = 0.06	1.3 (0.9–2.0); *p* = 0.13	**1.8 (1.1–3.0); *p* = 0.02**	1.3 (0.9–2.0); *p* = 0.16
Other salty snacks	**1.5 (1.4–1.6); *p* < 0.001**	1.0 (0.8–1.5); *p* = 0.67	1.0 (0.7–1.3); *p* = 0.79	1.3 (0.9–2.1); *p* = 0.18	1.1 (0.7–1.5); *p* = 0.78
Candies	**4.4 (4.0–4.9); *p* < 0.001**	1.0 (0.9–1.1); *p* = 0.80	1.1 (0.9–1.3); *p* = 0.32	1.2 (0.9–1.7); *p* = 0.18	1.2 (0.9–1.5); *p* = 0.26
Breakfast pastries	**3.4 (3.0–3.8); *p* < 0.001**	**0.6 (0.5–0.8); *p* = 0.001**	1.0 (0.7–1.3); *p* = 0.84	1.4 (0.9–2.0); *p* = 0.12	1.2 (0.7–2.1); *p* = 0.45
Cookies, pies, and cakes	**4.1 (3.7–4.6); *p* < 0.001**	1.0 (0.9–1.2); *p* = 0.87	1.0 (0.7–1.3); *p* = 0.76	1.0 (0.7–1.4); *p* = 0.84	0.9 (0.6–1.3); *p* = 0.65
Low or non-fat frozen dessert	**17.4 (13.1–23.0); *p* < 0.001**	0.9 (0.6–1.2); *p* = 0.46	**1.8 (1.0–3.3); *p* = 0.049**	0.9 (0.3–3.0); *p* = 0.92	2.0 (1.0–4.0); *p* = 0.06
Regular ice-cream and milkshakes	**16.7 (13.6–20.6); *p* < 0.001**	1.0 (0.8–1.2); *p* = 0.84	**1.4 (1.1–1.8); *p* = 0.002**	**0.6 (0.5–0.9); *p* = 0.006**	1.0 (0.7–1.5); *p* = 0.81
**Milk-based beverages**					
1.5% or non-fat milk	**28.4 (21.8–37.1); *p* < 0.001**	1.0 (0.9–1.2); *p* = 0.75	1.0 (0.7–1.3); *p* = 0.91	**1.5 (1.0–2.1); *p* = 0.03**	1.0 (0.7–1.4); *p* = 0.90
Regular or 3.0% milk	**18.5 (14.7–23.2); *p* < 0.001**	0.9 (0.8–1.1); *p* = 0.47	1.1 (0.9–1.5); *p* = 0.32	0.9 (0.6–1.2); *p* = 0.39	1.3 (0.9–1.9); *p* = 0.20
**Low-carb. drinks**					
Flavored water	**3.9 (3.4–4.5); *p* < 0.001**	1.2 (0.9–1.8); *p* = 0.25	**2.2 (1.1–4.5); *p* = 0.02**	**3.6 (1.5–8.7); *p* = 0.004**	**3.3 (1.6–6.5); *p* = 0.001**
Diet soda	**8.2 (6.8–9.8); *p* < 0.001**	**0.7 (0.5–0.97); *p* = 0.03**	1.5 (0.9–2.3); *p* = 0.09	1.8 (0.9–3.7); *p* = 0.11	**2.3 (1.3–4.0); *p* = 0.006**
**Fruit and vegetables**					
100% fruit juices	**5.0 (4.4–5.6); *p* < 0.001**	1.1 (0.9–1.3); *p* = 0.40	1.2 (0.9–1.5); *p* = 0.32	**1.7 (1.2–2.4); *p* = 0.003**	**1.5 (1.01–2.1); *p* = 0.04**
Vegetables	**1.7 (1.6–1.9); *p* < 0.001**	1.1 (0.97–1.2); *p* = 0.18	0.98 (0.8–1.2); *p* = 0.90	0.8 (0.6–1.1); *p* = 0.15	1.0 (0.8–1.2); *p* = 1.0
Fruit	**1.5 (1.4–1.6); *p* < 0.001**	1.0 (0.95–1.1); *p* = 0.40	0.9 (0.8–1.1); *p* = 0.37	**0.7 (0.6–0.9); *p* = 0.007**	1.0 (0.8–1.2); *p* = 0.84

*Obtained through three-level Poisson Models with random intercept and with students (level 1 units) nested into classroom (level 2 units) nested into schools (level 3 units). Values describe the difference expressed in terms of IRR with the relative 95% CI and -value, between covariates (environment, gender, class of age, and ethnic group) and outcomes in terms of weekly/daily consumption. In bold, IRRs statistically significant; only -values 0.05 were reported in bold.

### Snack consumption assessment

The BSQ also assessed the consumption of products that contain high-fat and/or high-sugar levels, such as potato chips and other salty snacks, candies, breakfast pastries, cookies, pies, cakes, frozen desserts, ice-cream, and milkshakes. Differences in the at school and out-of-school consumption were similar using both descriptive (Table 2) and multivariate analyses (Table 3). In particular, the higher consumption in the out-of-school setting ranged from 1.5 times in the case of “*other salty snacks*” (95% CI 1.4–1.6) to 17.4 times in the case of “*low or non-fat frozen dessert*” (95% CI 13.1–23.0). Females ate fewer breakfast pastries than males (IRR 0.6, 95% CI 0.5–0.8). Students aged (8–11) more often consumed low or non-fat frozen dessert (IRR 1.8, 95% CI 1.0–3.3) and regular ice-cream and milkshakes (IRR 1.4, 95% CI 1.1–1.8) than older students. Older students more commonly ate low-fat and regular potato chips and candies (IRR 2.1 and 1.8 respectively), but consumed regular ice-cream and milkshakes (IRR 0.6, 95% CI 0.5–0.9) less frequently than the younger ones. No ethnicity differences were observed with regard to salty and high-fat snack and dessert food consumption.

### Milk-based beverage consumption assessment

The next section on the BSQ assessed intake of low-fat milk, yogurt containing 1.5% fat, regular milk, and yogurt with 3% fat. In general, the median score of the weekly/daily distribution of consumption was higher in the out-of-school setting (one to four times per day) than in schools, as illustrated in Table 2 (descriptive statistics) and Table 3 (multivariate analysis). Students consumed more milk-based beverages in the out-of-school setting compared to the school setting (IRR 28.4 for 1.5% or non-fat milk and IRR 18.5 for regular or 3% milk). No differences were observed between gender, age, and ethnic groups, except for students aged >11 years who consumed more non-fat milk than the youngest students (IRR 1.5, 95% CI 1.0–2.1).

### Low-carbohydrate drink consumption assessment

The next section of the BSQ assessed flavored water drinks and diet drinks. These beverage categories typically contain artificial low-caloric sweeteners and thus classified as *non-obesogenic* beverages. Consumption estimates for these beverages were significantly different for at-school vs. out-of-school settings (Table 2). Additionally, consumption estimates differed by gender, age, and ethnic group (Table 3). In particular, students consumed flavored water and diet soda more frequently out of school than at school (IRRs 3.9 and 8.2, respectively). Females consumed diet soda slightly less often than males (IRR 0.7, 95% CI 0.5–0.97), students aged >8 years consumed more flavored water than their younger colleagues [IRRs 2.2 for students aged (8–11), 3.6 for students aged >11] and non-Caucasian students consumed low-carbohydrate beverages more frequently than Caucasians (IRRs 3.3 for flavored water and 2.3 for diet soda).

### Fruit and vegetable consumption assessment

The last three items of the BSQ examined the weekly intake of fresh fruit, 100% fruit juices, and vegetables. Interestingly, fruit consumption was low in the school setting, but not in the out-of-school setting (Table 2). Despite the fact that the schools in this study provided students with two fruit and one vegetable portion per day, the results from this study show that these foods were not consumed (Table 2). These result were upheld by the multivariate models, which estimated five times more frequent consumption of 100% fruit juices (95% CI 4.4–5.6) in the out-of-school setting compared to the school setting, while consumption frequency of vegetables and fruit was double to 1.5 times higher for out of school compared within school (IRR 1.7, 95% CI 1.6–1.9 and 1.5, IRR 1.5, 95% CI 1.4–1.6), respectively. Moreover, Caucasians consumed more fruit juices than non-Caucasian students (IRR 1.5, 95% CI 1.01–2.1). Students aged >11 years old consumed fruits less frequently than the youngest students (IRR 0.7, 95% CI 0.6–0.9), but also were more frequent consumers of fruit juices (IRR 1.7, 95% CI 1.2–2.4).

## Discussion

This cross-sectional survey of students in northern Italy examined students’ consumption of sweet and salty snacks and sugar-sweetened beverages in both the school and away from school environments. Importantly, the school districts included in the survey were those that had implemented policies to reduce access to these unhealthy foods during the school day. Our principal finding was that snacks and beverages were consumed out of school. While we are not evaluating the policy *per se* and we do not have pre-policy data, the finding in this report provides some optimism that the policies function as intended since we found negligible consumption of sugar-sweetened beverages and unhealthy snacks at school. These findings suggest that school-based policies to limit or deny access to unhealthy foods in the school environment are successful and may play an important role in promoting more healthful dietary patterns for school children. However, additional studies that compare the consumption of unhealthy foods among students attending schools with such policies to that of students attending schools without food policies would provide more definitive data on whether the interventions were successful in reducing the overall target food/beverage intakes.

One of our interesting findings was that there were differences in consumption of beverages and snacks by gender and age. Some “*obesogenic”* dietary behaviors tended to worsen in males and older students. Older students may have had more opportunities to be exposed to unhealthful food than their younger peers, especially in the milieu of out-of school uncontrolled activities. This scenario was also highlighted by *Third School Nutrition Dietary Assessment Study 2004–2005* (SNDA-III) data analysis (29) where authors found that students’ access to school stores and snack bars, with the subsequent consumption of sugar-sweetened beverages, was significantly increased in the secondary schools.

Similarly, a recent survey that was commissioned by EFSA to the Consortium Nomisma Aretè, with the objective of gathering consumption data for “energy drinks” in specific consumption groups strongly noticed that more than 95% of children and adolescents consume energy drinks out-of-the school (30). This may be because students could have more opportunities outside the school to consume unhealthy food (i.e., vending machines that are frequently located in the halls of sports centers or in other places where students spend their spare time) or students could be encouraged by parental behavior to eat more food, including unhealthy food, than would meet their actual nutritional needs. This finding is also consistent with the findings of “*OKKIO alla Salute*,” the national surveillance program on overweight and obesity for students between 6 and 10 years of age. The last survey, completed in 2012, revealed that 33% of Italian students were overweight or obese (31) and that the impact and consequences of the phenomenon were not perceived by parents who, on the contrary, seemed to overestimate students’ nutritional needs (32)^.^

For younger students, the snacks and beverages consumed at school were probably brought from home or purchased on the way to school since purchase was not possible at school. This is consistent with a study conducted on a cohort of elementary school students, which found that sugar-sweetened beverages consumed at school were brought from home (29).

The Italian Ministry of Health has funded a number of health promotion projects in order to prevent overweight and obesity in childhood. These projects involved teachers and students with the main aim of improving dietary-related behaviors in schools. In addition, since 2002, the Unit of Public Health and Disease Prevention of the Veneto Region has developed programs of health surveillance, health education, and obesity prevention through healthy eating in the schools of the Region (26, 33, 34). However, even though good progress has been made in this field, much remains to be done. For example, schools are still not able to guarantee a good consumption of fruit and vegetables, since a strong difference in the consumption of these products between the two explored settings has been observed in the current study, with more frequent fruit/vegetable consumption out of school. The guidelines for healthy eating suggest consuming five portions of fruit and vegetables per day (*five a day*) (35). This recommendation was apparently not followed by students enrolled in the present study since they reported infrequent consumption of fruits and vegetables, thus may fail to meet their vitamin needs (36, 37). Students were expected to eat fruit and vegetables with a comparable frequency at school and out of school, as they normally should consume three of the five suggested daily portions during the school time. It is possible that the low vegetable intake is due to the lower palatability or lower familiarity of vegetable-based dishes served at school compared to those served at home. One solution would be to revise school menus, with an emphasis on provision of sufficient fruits and vegetables.

Many studies have associated parental education level with the frequency of students’ consumption of high-fat and high-sugar foods (38, 39), suggesting that children of parents with a low educational level may be at higher risk of unhealthy eating. Education might produce an important socio-economic influence on health-related behaviors as it may increase the use of health-related information and raise the level of awareness of food-related concerns (40). However, to our knowledge, few obesity prevention programs aimed at improving parents’ health literacy have been implemented for now in Italy.

Thus, future interventions should include the design and implementation of appropriate information campaigns aimed specifically at the out-of school environment, including the family.

Sports participation, social recognition of peers, and socio-cultural pressures also play central roles in both students’ and adolescents’ preference construction, including food choices. Whether, and how, these factors can affect eating habits and lifestyles will be the subject of further investigations from which more targeted preventive interventions aimed at unhealthy food consumption practices will be developed.

## Author Contributions

CL conceived and designed the research, acquired and interpreted the data, drafted the work; VC analyzed the data; MN designed and interpreted the data, revised the work critically; VG interpreted the data; IA conceived the research and interpreted the data; AR conceived and designed the research, interpreted the data, and drafted the work.

## Conflict of Interest Statement

The authors declare that the research was conducted in the absence of any commercial or financial relationships that could be construed as a potential conflict of interest.

## Footnotes

^1^http://www.fruttanellescuole.gov.it

^2^http://www.educazionedigitale.it/prodotto/forchetta-e-scarpetta/
